# Coccidioidomycosis and COVID-19 Co-Infection, United States, 2020

**DOI:** 10.3201/eid2705.204661

**Published:** 2021-05

**Authors:** Alexandra K. Heaney, Jennifer R. Head, Kelly Broen, Karen Click, John Taylor, John R. Balmes, Jon Zelner, Justin V. Remais

**Affiliations:** University of California, Berkeley, California, USA (A.K. Heaney, J.R. Head, K. Click, J. Taylor, J.R. Balmes, J.V. Remais);; University of Michigan, Ann Arbor, Michigan, USA (K. Broen, J. Zelner);; University of California, San Francisco, California, USA (J.R. Balmes)

**Keywords:** COVID-19, coccidioidomycosis, co-infections, diagnosis, risk factors, SARS-CoV-2, respiratory infections, severe acute respiratory syndrome coronavirus 2, coronavirus disease, zoonoses, viruses, coronaviruses, fungi, California, Arizona, United States, Coccidioides

## Abstract

We review the interaction between coronavirus disease (COVID-19) and coccidioidomycosis, a respiratory infection caused by inhalation of *Coccidioides* fungal spores in dust. We examine risk for co-infection among construction and agricultural workers, incarcerated persons, Black and Latino populations, and persons living in high dust areas. We further identify common risk factors for co-infection, including older age, diabetes, immunosuppression, racial or ethnic minority status, and smoking. Because these diseases cause similar symptoms, the COVID-19 pandemic might exacerbate delays in coccidioidomycosis diagnosis, potentially interfering with prompt administration of antifungal therapies. Finally, we examine the clinical implications of co-infection, including severe COVID-19 and reactivation of latent coccidioidomycosis. Physicians should consider coccidioidomycosis as a possible diagnosis when treating patients with respiratory symptoms. Preventive measures such as wearing face masks might mitigate exposure to dust and severe acute respiratory syndrome coronavirus 2, thereby protecting against both infections.

Persons with coronavirus disease (COVID-19) can have a wide range of symptoms, including cough, difficulty breathing, and fatigue (*1*). These symptoms are also common among patients with coccidioidomycosis (*2*), a primarily pulmonary disease caused by inhalation of *Coccidioides*, a soil-dwelling dimorphic fungi. These spores spread through the air, especially through wind erosion in dusty environments and dirt disrupting activities such as digging or construction. *Coccidioides* spores are found in hot and arid environments, including much of the southwestern United States, where coccidioidomycosis incidence has been increasing. Since 2016, California has recorded its highest incidences of coccidioidomycosis (*3*,*4*).

We reviewed epidemiologic and clinical literature on coccidioidomycosis and COVID-19 to identify subpopulations that might be at risk for co-infection and severe disease. We discuss how the COVID-19 pandemic might affect coccidioidomycosis diagnosis, surveillance, and clinical management. We also evaluate evidence that co-infection might contribute to severe COVID-19 or reactivation of latent *Coccidioides* infection. Our study informs healthcare providers, policymakers, and populations in regions to which coccidioidomycosis is endemic on potential interactions between this disease and COVID-19, encouraging protective measures and prompt diagnosis.

## Methods

We searched peer-reviewed journals on PubMed, Google Scholar, Scopus, and Web of Science; preprints posted on medRxiv and bioRxiv; and reports from state health departments and correctional agencies for articles on risk factors for infection and disease severity, diagnosis, surveillance, and preventive measures for coccidioidomycosis and COVID-19. We assessed titles and abstracts for relevance to the risk factors, diagnostic issues, and complications of coccidioidomycosis and COVID-19 co-infections. We conducted searches published during April–December 2020 and did not exclude articles on the basis of publication date. We identified other relevant publications by backward citation searching. We analyzed 116 peer-reviewed articles, 4 preprints, and 28 reports.

## Possible Risk Factors for Coccidioidomycosis and COVID-19

**Figure 1:**
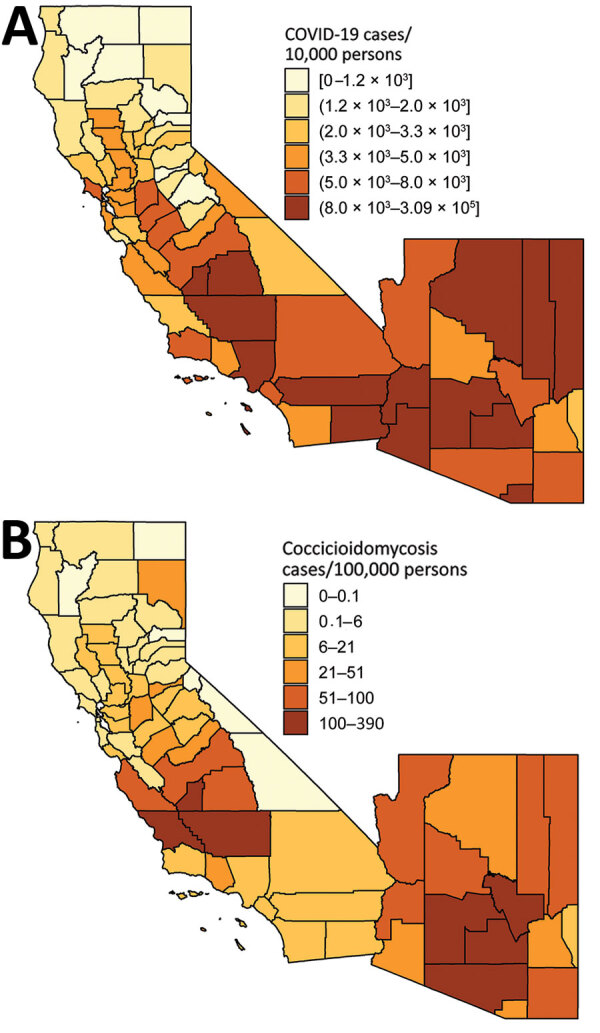
County-level incidence of (A) coronavirus disease (COVID-19) in 2020 and (B) coccidioidomycosis in 2019, California and Arizona. COVID-19 incidence reflects cumulative case count as of August 14, 2020 (*5*). Coccidioidomycosis incidence reflects annual incidence in 2019 (*6*,*7*). Shading indicates levels of incidence. Brackets indicate inclusive bounds; parentheses indicate exclusive bounds.

**Figure 2:**
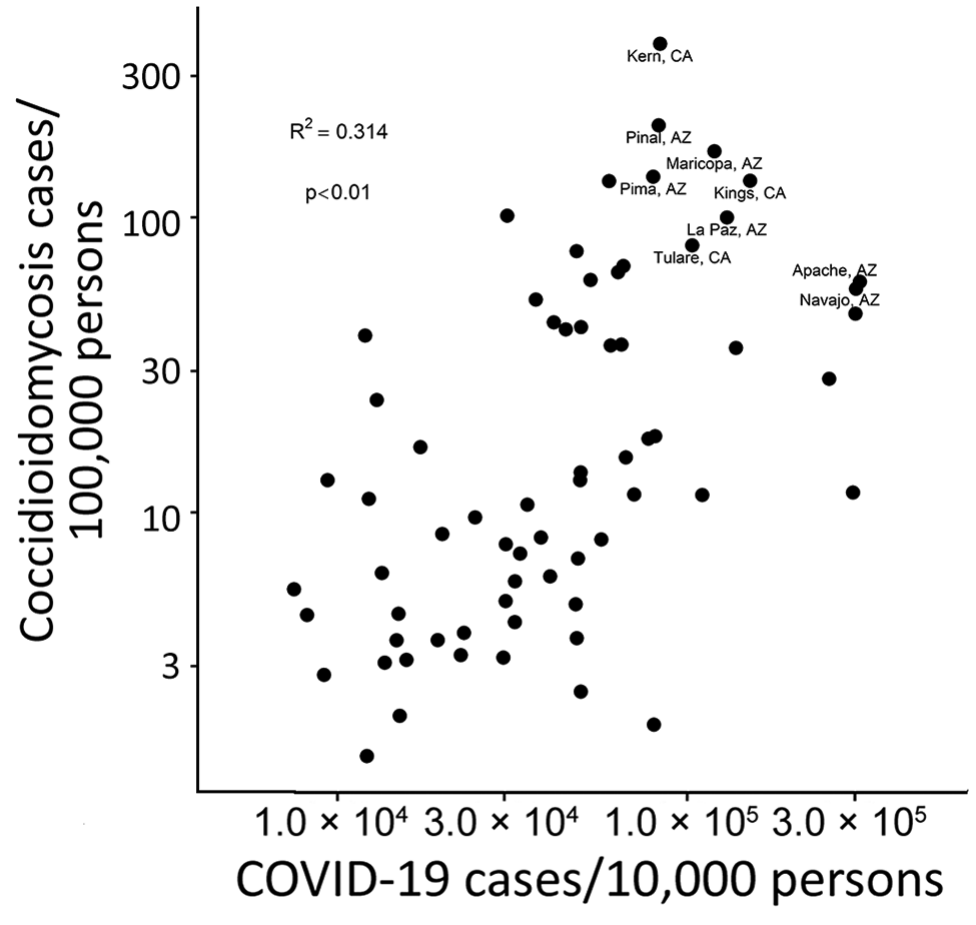
Scatterplot of county-level incidence of COVID-19 in 2020 and coccidioidomycosis in 2019, California and Arizona. R^2^ = 0.259; p<0.01.

COVID-19 and coccidioidomycosis share certain risk factors for exposure, potentially increasing the risk for co-infection. In California and Arizona, the 2 states with the highest number of reported coccidioidomycosis cases, substantial overlap exists between county-level incidence of COVID-19 in 2020 and coccidioidomycosis in 2019 (Figures 1, 2).

### Occupational Risks

Certain occupations pose increased risk for coccidioidomycosis. Because soil disruption and dusty environments promote dispersal of *Coccidioides* spores, coccidioidomycosis outbreaks frequently occur among workers in the construction and agricultural sectors (*8*,*9*,*10*). Of 47 coccidioidomycosis outbreaks reported during 1940–2015, a total of 25 (53%) were associated with occupational exposure, including 15 (60%) that were related to construction (*11*). An analysis of workers’ compensation claims found that the incidence of coccidioidomycosis related to occupational exposure nearly quadrupled in California during 2000–2006, the highest rates seen among construction and agricultural workers (*12*).

Continued in-person work within the construction and agricultural sectors, which are considered essential occupations, also increases risk for COVID-19. In the United States, an estimated 8% of construction workers have had workplace exposure to the causative agent of COVID-19, severe acute respiratory syndrome coronavirus 2 (SARS-CoV-2), at least monthly, and nearly 60% of the construction labor force has >1 risk factor for severe COVID-19 (*13*, *14*). Agricultural workers might also have heightened risk for COVID-19 because of high workforce turnover, shared transportation, and overcrowded living quarters that are often shared with other workers, multigenerational families, or both (*15*–*19*).

### Incarcerated Populations

Incarcerated persons have a high risk for exposure to *Coccidioides* spores and SARS-CoV-2. Prisons and other facilities, such as immigration detention centers, are often in isolated areas with high environmental dust concentrations that can place inmates at higher risk for *Coccidioides* infection (Appendix). In addition, crowding, unsanitary conditions, and poor ventilation in carceral environments contributes to the rapid spread of communicable respiratory diseases like COVID-19 (*20*). Researchers have documented COVID-19 outbreaks among fire-fighting crews composed of incarcerated persons (*21*); similarly, researchers documented 7 coccidioidomycosis outbreaks among such fire-fighting crews during 2000–2017 (*22*). During 1940–2015, a total of 5 (11%) reported coccidioidomycosis outbreaks were among incarcerated populations (*11*). During 2007–2011, a total of 19% of coccidioidomycosis cases in California were among incarcerated persons (*23*). More than 25% of California Department of Corrections and Rehabilitation facilities, including Lompoc Prison Complex (Lompoc, CA, USA), where a COVID-19 outbreak infected >1,000 persons (*24*), are in regions with high coccidioidomycosis incidence (*25*).

Researchers have documented several outbreaks of COVID-19 in carceral facilities (Appendix). During January 21–April 21, 2020, a total of 82% of reporting state and territorial health department jurisdictions reported confirmed COVID-19 cases among incarcerated or detained persons (including 4,893 reported cases and 88 deaths) or staff members (including 2,778 reported cases and 15 deaths) (*26*). COVID-19 outbreaks affecting >1,000 persons have occurred among incarcerated persons and staff working at carceral facilities in states from California to New York (Appendix).

### Racial and Ethnic Minorities

Substantial racial and ethnic disparities exist in COVID-19 and coccidioidomycosis infection rates. Persons of Black and Latino heritage are at heightened risk for these infections. In California as of February 2021, Latino persons comprise 39% of the total population but account for 55% of COVID-19 cases (*27*). In the United States, COVID-19 incidence and death rates in counties with predominantly Black populations are significantly higher than in counties with predominantly white populations (*28*). In addition, Latino persons comprise 39% of the California population but 47% of its coccidioidomycosis patients; in the same state, non-Hispanic Black persons comprise 6% of the population but 9% of coccidioidomycosis patients (*3*).

Numerous societal inequities (including racism and discrimination, economic and educational disadvantages, and lack of healthcare access) contribute to higher pathogen exposure and infection rates among Black and Latino populations (*29*). In the context of the COVID-19 pandemic, social distancing might be more difficult for persons of low socioeconomic status because of their overrepresentation in essential occupations, elevated risk of living in densely populated homes and neighborhoods, and higher numbers of multigenerational households (*15*–*19*). For example, 55% of Latino and 48% of Black persons work in essential jobs, compared with 35% of White persons (*30*). Disparities in coccidioidomycosis rates might also be caused by the disproportionate numbers of Black and Latino persons who are incarcerated or work in occupations with high exposure risk. More than 50% of farm laborers, agricultural workers, and construction workers in California are Latino (*31*,*32*). In addition, Black and Latino persons are overrepresented in carceral facilities: in California, Black persons comprise 27% and Latino persons comprise 41% of jail and prison populations (*33*). 

### Exposure to Particulate Matter

Persons living in environments with high concentrations of dust, which is a major constituent of particulate matter <10 μm or <2.5 μm in diameter, might be at elevated risk for infection with *Coccidioides* and SARS-CoV-2 and severe illness from COVID-19. Exposure to particulate matter is a risk factor for illness and death from viral respiratory infections, including COVID-19 (Appendix). Exposure to outdoor particulate air pollution is also associated with *Coccidioides* infection because mineral dust can mobilize airborne spores (*34*,*35*). Coccidioidomycosis outbreaks have been linked to dust plumes generated by military exercises, agriculture, construction, archeology excavations, windstorms, and landslides (*36*–*43*). For example, in an outbreak affecting 89 persons at a solar farm, persons who reported being in a dust cloud had ≈6 times the odds of symptomatic coccidioidomycosis than those who were not in the dust cloud. Wetting the dirt before soil-disrupting activities, a common practice to reduce dust, decreased the odds of symptomatic infection by 58% (*44*). Because COVID-19 control measures encourage the use of outdoor spaces, persons might have increased exposures to mineral dust and other air pollutants during the pandemic. 

## Co-Circulation with SARS-CoV-2 Hampering Coccidioidomycosis Diagnosis

The diagnosis of coccidioidomycosis in areas with community transmission of COVID-19 might be challenging because the diseases cause similar symptoms, which might exacerbate existing delays in coccidioidomycosis diagnosis and treatment. Without antifungal treatment, coccidioidomycosis patients are at risk for severe illness, including disseminated disease, and for death (*45*). Promptly administering of antifungal treatments reduces unnecessary use of antimicrobial drugs and resolves symptoms more effectively (*45*). In addition, early case management, including assessing of risk factors for severity, regular follow-up visits to monitor symptoms, regular testing to check antibody titer levels, and physical therapy, is crucial to mitigating severe disease (*46*).

One reason for the underdiagnosis of coccidioidomycosis is low testing rates. For instance, a study in Tucson, Arizona, estimated that 15%–44% of community-acquired pneumonia cases could be attributed to coccidioidomycosis (*47*), but only 2%–13% of community-acquired pneumonia cases were tested for coccidioidomycosis (*48*). Valdivia et al. (*47*) found that half of patients had >2 clinic visits before being tested for coccidioidomycosis. Low sensitivities of coccidioidomycosis tests might further contribute to delays in diagnosis (Appendix). Given such diagnostic constraints, the median time between seeking healthcare and coccidioidomycosis diagnosis was estimated to be 23 days in Arizona (*49*).

The COVID-19 pandemic might contribute to further delays in coccidioidomycosis diagnosis. Both diseases can cause dry cough, muscle aches, headache, fatigue, and difficulty breathing; however, patients with COVID-19 tend to have a more acute progression of symptoms than those with coccidioidomycosis (*50*–Reference 54 in Appendix). Although pulmonary specialists and primary care physicians in regions to which coccidioidomycosis is endemic are probably aware of the diagnosis and treatment of this fungal infection, physicians in other regions might be less familiar with the diagnosis. Attributing coccidioidomycosis symptoms to COVID-19, whether presumed or laboratory-confirmed, might preclude coccidioidomycosis diagnosis in patients with monoinfections or co-infections. In addition, underutilization of healthcare services during the COVID-19 pandemic might result in further delays in the testing and diagnosis of coccidioidomycosis (Reference 55 in Appendix).

## Risk Factors for Severe Disease

Although most cases of coccidioidomycosis or COVID-19 are mild respiratory illnesses, either infection can cause severe disease and death (Appendix). Risk factors associated with severe coccidioidomycosis or COVID-19 often overlap, prompting concerns of elevated death rates associated with co-infections or serial infections. Patients with SARS-CoV-2 and *Coccidioides* co-infection might be at higher risk for severe disease; however, whether synergistic effects might exist requires further data. Overlapping risk factors associated with severe disease caused by coccidioidomycosis or COVID-19 include older age, diabetes mellitus, immunosuppression, Black/African American ancestry, and smoking (References 56–70 in Appendix). Although the long-term pulmonary effects of COVID-19 remain unknown, early data suggest that the virus might cause lung damage (Reference 71 in Appendix), resulting in elevated long-term risk for severe coccidioidomycosis.

### Age

 Older persons have heightened risk for severe disease caused by either infection. In the United States, 62% of COVID-19 hospitalizations and 80% of deaths were among patients >65 years of age (Reference 72 in Appendix). Similarly, older persons, especially those >65 years of age, with coccidioidomycosis have higher risk for severe pulmonary disease. Rates of coccidioidomycosis-associated death increase with age. These trends might be partially explained by the higher prevalence among older adults of preexisting conditions and immunosuppression, which are risk factors for severe COVID-19 and coccidioidomycosis (References 56–64 in Appendix).

### Diabetes

Diabetes is also associated with severe progression of either disease (References 56,63–68 in Appendix). A study of COVID-19 patients found that those with diabetes had a higher risk for severe pneumonia and organ damage (Reference 73 in Appendix). The study also showed that patients with diabetes were more susceptible to a SARS-CoV-2–induced cytokine storm, which can cause rapid deterioration and death (Reference 73 in Appendix). In addition, patients with diabetes are more likely to have relapsing coccidioidomycosis (risk ratio [RR] 3.39, 95% CI 1.65–6.46) or cavitary lung disease (RR 2.94, 95% CI 1.63–4.90) than those without diabetes (Reference 74 in Appendix). Furthermore, among coccidioidomycosis patients with diabetes, those with higher serum glucose levels are more likely to have disseminated coccidioidomycosis, the most severe form of the disease, than those with lower levels (Reference 74 in Appendix). The exact mechanisms through which diabetes influences the progression of coccidioidomycosis and COVID-19 are not well understood but might be related to impaired innate and adaptive cellular immunity (especially T-cell function) or the effects of a hyperglycemic environment on microorganism virulence (Reference 75 in Appendix).

### Immunosuppression

Although immunosuppressive steroids such as dexamethasone have reduced inflammatory lung damage in patients with severe COVID-19 (Reference 76 in Appendix), emerging evidence suggests that persons with a history of prolonged immunosuppression might be at higher risk for severe COVID-19. A study of 17 million adults in the United Kingdom found higher risks for death among COVID-19 patients who have hematologic malignancies, who are taking immunosuppressant drugs for organ transplants, or who have other causes of immunosuppression (Reference 77 in Appendix). Immunosuppressed patients with cancer or solid organ transplants might be at higher risk for severe COVID-19 (Reference 78 in Appendix). Coccidioidomycosis patients with suppressed immune responses, such as patients with hematologic malignancies, HIV, or organ transplants, also have higher risk for disseminated disease (References 61–63 in Appendix).

### Black/African American Ancestry

Black persons have higher rates of severe COVID-19 and disseminated coccidioidomycosis than do White persons. Growing evidence indicates higher risk for severe COVID-19–associated disease and death among Black than White persons living in the United States (Appendix). A study of coccidioidomycosis in military personnel found dissemination rates to be 10 times higher among Black than White persons (Reference 79 in Appendix). Similarly, a study in Kern County, California, found that patients with disseminated coccidioidomycosis were 4.6 times more likely to be Black than patients with mild disease (Reference 56 in Appendix). The observed racial and ethnic disparities in severe COVID-19 and coccidioidomycosis are probably driven by structural inequities that systematically disadvantage persons of color in the forms of reduced healthcare access and exposure to environmental stressors that increase risk for conditions such as diabetes, obesity, and hypertension, which are associated with severe disease (*29*). For coccidioidomycosis, whether any biological basis for this association exists is unclear but might be related to immunogenic differences in T-cell function (References 56,69,70 in Appendix).

### Smoking

Recent history of cigarette smoking has been linked to higher risk for severe disease from either infection. A systematic review and meta-analysis found that smokers with COVID-19 were significantly more likely (RR 2.4, 95% CI 1.43–4.04) to be admitted to an intensive care unit, need mechanical ventilation, or die compared with nonsmokers (Reference 80 in Appendix). A case–control study in Kern County found that patients with severe or disseminated coccidioidomycosis were more likely to have smoked cigarettes in the previous 6 months compared with patients with mild coccidioidomycosis (Reference 56 in Appendix).

## Possible Effects of Co-Infection on Disease Progression

### Severe COVID-19

Underlying respiratory illness is a major risk factor for severe COVID-19 (References 60,64 in Appendix). The Centers for Disease Control and Prevention reported that among COVID-19 patients in the United States with available data on concurrent conditions, 9.2% had a chronic lung disease such as chronic obstructive pulmonary disease, asthma, or emphysema; chronic lung disease was the most common concurrent condition after diabetes (Reference 81 in Appendix). The prevalence of chronic lung disease is higher among hospitalized patients (15%) and highest among patients in the intensive care unit (21%) (Reference 81 in Appendix). Several studies of COVID-19 patients in China have also shown elevated rates of death and severe disease among those with underlying chronic respiratory conditions (References 64,82,83 in Appendix). Acute coccidioidomycosis is often self-limiting, but ≈3%–5% of patients have a chronic pulmonary form of the illness (References 84,85 in Appendix). The evidence that chronic lung disease increases risk for severe COVID-19 suggests that patients with chronic pulmonary coccidioidomycosis might be predisposed to severe COVID-19.

### Coccidioidomycosis Reactivation

Infection with COVID-19 might reactivate disease in a coccidioidomycosis patient whose illness has progressed to a chronic but inactive state. After an initial *Coccidioides* infection resolves, the fungus can remain in the lungs in a latent state and become reactivated under certain conditions (References 86–93 in Appendix). Coccidioidomycosis reactivation has been reported among pregnant women, especially those who previously had disseminated coccidioidomycosis (Reference 94 in Appendix). Patients with organ transplants, which usually require immunosuppressive medications, also have higher rates of coccidioidomycosis reactivation (References 87–92 in Appendix). SARS-CoV-2 infection has been associated with immune dysregulation, including lymphopenia (Reference 95 in Appendix), which might lower the host's ability to regulate *Coccidioides* infection (Reference 96 in Appendix). Although no studies have reported coccidioidomycosis reactivation in COVID-19 patients as of February 2021, emerging evidence suggests that COVID-19 infection might accelerate the reactivation of latent tuberculosis (L. Pathak, unpub. data, https://www.biorxiv.org/content/10.1101/2020.05.06.077883v2). In addition, dexamethasone, a medication recommended for patients with severe COVID-19, increases the risk for severe coccidioidomycosis (References 97,98 in Appendix).

## Areas for Future Research

### Cloth Masks

Although cloth masks are a critical control method for COVID-19 (Appendix), studies have not examined the efficacy of cloth masks for filtering *Coccidioides* arthroconidia. At 2–5 μm in diameter, *Coccidioides* arthroconidia are substantially larger than SARS-CoV-2; this size difference might lead to differing levels of filtration effectiveness (References 99,100 in Appendix). One study found that cloth masks containing tightly woven cottons can filter 98% of particles in the 300 nm–6 µm range (Reference 101 in Appendix), yet such results are difficult to extrapolate to specific particles such as *Coccidioides* arthroconidia (Reference 102 in Appendix). It is also difficult to extrapolate results to other cloth masks, which vary widely in their filtration properties. Furthermore, leakage from improperly fitting masks can reduce efficacy of particle filtration by up to 50% (Reference 101 in Appendix). The effects of leakage on disease prevention might differ on the basis of infectious dose; although a single *Coccidioides* spore might confer infection, the infectious dose of SARS-CoV-2 is probably higher. California therefore requires employers with worksites in regions to which coccidioidomycosis is endemic to provide respiratory protection filters rated at least N95 to workers if dust cannot be controlled; no mask recommendation exists for the general public (Reference 103 in Appendix).

### Climate

Transmission of SARS-CoV-2 and *Coccidioides* spores might be influenced by climatic conditions, such as temperature and humidity, that can affect pathogen survival and transport. For example, high humidity can suppress aerosol transmission of respiratory pathogens such as influenza and respiratory syncytial virus (References 104–110 in Appendix). Early research in Wuhan, China, suggested that SARS-CoV-2 might be transmitted more efficiently in less humid environments (References 111–113; in Appendix, W. Luo, unpub. data, https://www.medrxiv.org/content/10.1101/2020.02.12.20022467v1). Although the influence of temperature and other climatic conditions on transmission and seasonality of SARS-CoV-2 currently might be outweighed by the large size of the susceptible population, the introduction of a vaccine could result in patterns of population immunity that enable climate to play a larger moderating role (Reference 114 in Appendix). Because relative humidity plays a major role in regulating atmospheric dust concentrations, high atmospheric moisture can limit the dispersal of *Coccidioides* spores, potentially suppressing coccidioidomycosis transmission. For example, under wind conditions strong enough to mobilize dust, increases in relative humidity were associated with decreasing atmospheric dust concentrations (Reference 115 in Appendix).

### Disparities in Surveillance

 The extent of socioeconomic, demographic, racial, and other disparities in COVID-19 and coccidioidomycosis is probably greater than reflected in administrative data sources. For example, analyses from hard-hit regions have indicated that high rates of excess death probably reflect a large burden of unreported SARS-CoV-2 infection (Reference 116; in Appendix, J. Felix-Cardoso, unpub. data, https://www.medrxiv.org/content/10.1101/2020.04.28.20083147v1). Although testing coverage for SARS-CoV-2 is increasing, infections will probably continue to be undercounted in certain regions and populations because of factors such as disparate healthcare access, reagent shortages, and varied willingness to get tested. Undocumented or migrant farmworkers at high risk for exposure to *Coccidioides* spores are mostly uninsured, ineligible for healthcare benefits, or unable to afford healthcare (References 117,118 in Appendix). The disparities seen in rates of illness and death caused by COVID-19 and coccidioidomycosis might have many contributing factors, including barriers to affordable, high-quality, and accessible healthcare; occupational exposures; mass incarceration; residential segregation; discrimination; and differential rates of concurrent conditions. Understanding these disparities is critical for attracting the attention and resources needed to remedy inequities in exposures, care-seeking, and illness and death caused by coccidioidomycosis and COVID-19.

## Conclusions

Public health professionals, healthcare providers, and populations in areas to which coccidioidomycosis is endemic should be aware of the overlap in risk factors for coccidioidomycosis and COVID-19. Because prompt diagnosis is critical for effective management of coccidioidomycosis and the COVID-19 pandemic might exacerbate existing delays, healthcare professionals should know how to identify these diseases and potential co-infection. Agricultural and construction workers, firefighters, Black and Latino persons, persons with diabetes, elderly persons, incarcerated persons, and migrant or undocumented farmworkers might be at increased risk for coccidioidomycosis and COVID-19. Employers and public health officials should mitigate exposure to dust and SARS-CoV-2 by promoting the use of face masks and social distancing practices.

Appendix Further information on results and additional references in a study of coccidiomycosis and COVID-19 co-infection.

## References

[1] Goyal P, Choi JJ, Pinheiro LC, Schenck EJ, Chen R, Jabri A, et al. Clinical characteristics of Covid-19 in New York City. N Engl J Med. 2020;382:2372–4. 10.1056/NEJMc2010419 32302078 PMC7182018

[2] Blair JE, Chang YHH, Cheng MR, Vaszar LT, Vikram HR, Orenstein R, et al. Characteristics of patients with mild to moderate primary pulmonary coccidioidomycosis. Emerg Infect Dis. 2014;20:983–90. 10.3201/eid2006.131842 24865953 PMC4036774

[3] California Department of Public Health. Epidemiological summary of coccidioidomycosis in California, 2018. 2019 [cited 2020 May 18]. https://www.cdph.ca.gov/Programs/CID/DCDC/CDPH%20Document%20Library/CocciEpiSummary2018.pdf

[4] Centers for Disease Control and Prevention. Valley fever (coccidiomycosis) statistics. 2020 [cited 2020 May 14]. https://www.cdc.gov/fungal/diseases/coccidioidomycosis/statistics.html

[5] USAFacts. US coronavirus cases and deaths by state. 2020 [cited 2020 May 21]. https://usafacts.org/visualizations/coronavirus-covid-19-spread-map

[6] California Department of Public Health. Coccidioidomycosis in California provisional monthly report: January–April 2020. 2020 [cited 2020 May 28]. https://www.cdph.ca.gov/Programs/CID/DCDC/CDPH%20Document%20Library/CocciinCAProvisionalMonthlyReport.pdf

[7] Arizona Department of Health Services. Valley fever 2019 annual report. 2021 [cited 2021 Feb 24]. https://www.azdhs.gov/documents/preparedness/epidemiology-disease-control/valley-fever/reports/valley-fever-2019.pdf

[8] Levan NE, Huntington RW. Primary cutaneous coccidioidomycosis in agricultural workers. Arch Dermatol. 1965;92:215–20. 10.1001/archderm.92.3.215 14329224

[9] Schmelzer LL, Tabershaw IR. Exposure factors in occupational coccidioidomycosis. Am J Public Health Nations Health. 1968;58:107–13. 10.2105/ajph.58.1.107 PMC1228046 5688736

[10] Johnson WM. Occupational factors in coccidioidomycosis. J Occup Med. 1981;23:367–74. 7017083

[11] Freedman M, Jackson BR, McCotter O, Benedict K. Coccidioidomycosis outbreaks, United States and worldwide, 1940–2015. Emerg Infect Dis. 2018;24:417–23. 10.3201/eid2403.170623 29460741 PMC5823332

[12] Das R, McNary J, Fitzsimmons K, Dobraca D, Cummings K, Mohle-Boetani J, et al. Occupational coccidioidomycosis in California: outbreak investigation, respirator recommendations, and surveillance findings. J Occup Environ Med. 2012;54:564–71. 10.1097/JOM.0b013e3182480556 22504958

[13] Baker MG, Peckham TK, Seixas NS. Estimating the burden of United States workers exposed to infection or disease: A key factor in containing risk of COVID-19 infection. PLoS One. 2020;15:e0232452. 10.1371/journal.pone.0232452 32343747 PMC7188235

[14] Brown S, Brooks R, Dong XS. Coronavirus and health disparities in construction. The Center for Construction Research and Training. 2020 [cited 2020 Oct 16]. https://www.cpwr.com/wp-content/uploads/publications/DataBulletin-May2020.pdf

[15] Acs G, Loprest PJ. Job differences by race and ethnicity in the low-skill job market. The Urban Institute. 2009 [cited 2020 Jun 15]. https://www.urban.org/sites/default/files/publication/30146/411841-Job-Differences-by-Race-and-Ethnicity-in-the-Low-Skill-Job-Market.PDF

[16] Murray CJL, Kulkarni SC, Michaud C, Tomijima N, Bulzacchelli MT, Iandiorio TJ, et al. Eight Americas: investigating mortality disparities across races, counties, and race-counties in the United States. PLoS Med. 2006;3:e260. 10.1371/journal.pmed.0030260 16968116 PMC1564165

[17] Centers for Disease Control and Prevention. Communities, schools, workplaces, and events. 2020 [cited 2020 Oct 16]. https://www.cdc.gov/coronavirus/2019-ncov/community/guidance-agricultural-workers.html

[18] Arcury TA, Weir M, Chen H, Summers P, Pelletier LE, Galván L, et al. Migrant farmworker housing regulation violations in North Carolina. Am J Ind Med. 2012;55:191–204. 10.1002/ajim.22011 PMC3708262 22237961

[19] Quandt SA, Brooke C, Fagan K, Howe A, Thornburg TK, McCurdy SA. Farmworker housing in the United States and its impact on health. New Solut. 2015;25:263–86. 10.1177/1048291115601053 26320122

[20] Simpson PL, Simpson M, Adily A, Grant L, Butler T. Prison cell spatial density and infectious and communicable diseases: a systematic review. [Erratum in: BMJ Open. 2020;10: e026806corr1]. BMJ Open. 2019;9:e026806. 10.1136/bmjopen-2018-026806 31340959 PMC6661645

[21] Sabalow R, Pohl J. California severely short on firefighting crews after COVID-19 lockdown at prison camps. The Sacramento Bee. 2020 [cited 2020 Aug 18]. https://www.sacbee.com/news/california/fires/article243977827.html

[22] Lucas KD, Wheeler C, Mohle-Boetani JC. Coccidioidomycosis outbreaks among inmate wildland firefighters in California. Proceedings of the 63rd Coccidioidomycosis Study Group Annual Meeting; 2019 Apr 5–6. Sacramento, CA, USA.

[23] MacLean M. Epidemiology of coccidioidomycosis—15 California counties, 2007–2011. 2014 [cited 2020 May 28]. https://www.vfce.arizona.edu/sites/vfce/files/the_epidemiology_of_coccidioidomycosis_collaborative_county_report.pdf

[24] The New York Times. Coronavirus in the U.S.: latest map and case count. 2020 [cited 2020 May 28]. https://www.nytimes.com/interactive/2020/us/coronavirus-us-cases.html?auth=login-email&login=email

[25] Prison Law Office. Valley fever and CDCR housing. 2019 [cited 2020 May 28]. https://prisonlaw.com/wp-content/uploads/2019/04/Valley-Fever-info-April-2019.pdf

[26] Wallace M, Hagan L, Curran KG, Williams SP, Handanagic S, Bjork A, et al. COVID-19 in correctional and detention facilities—United States, February–April 2020. MMWR Morb Mortal Wkly Rep. 2020;69:587–90. 10.15585/mmwr.mm6919e1 32407300

[27] California Department of Public Health. COVID-19 race and ethnicity data. 2020 [cited 2020 Jun 15]. https://www.cdph.ca.gov/Programs/CID/DCDC/Pages/COVID-19/Race-Ethnicity.aspx

[28] Moore JT, Ricaldi JN, Rose CE, Fuld J, Parise M, Kang GJ, et al.; COVID-19 State, Tribal, Local, and Territorial Response Team. Disparities in incidence of COVID-19 among underrepresented racial/ethnic groups in counties identified as hotspots during June 5–18, 2020—22 states, February–June 2020. MMWR Morb Mortal Wkly Rep. 2020;69:1122–6. 10.15585/mmwr.mm6933e1 32817602 PMC7439982

[29] Zelner J, Trangucci R, Naraharisetti R, Cao A, Malosh R, Broen K, et al. Racial disparities in COVID-19 mortality are driven by unequal infection risks. Clin Infect Dis. 2020;72:e88–95. 10.1093/cid/ciaa1723 PMC7717213 33221832

[30] Thomason S, Bernhardt A. Front-line essential jobs in California: a profile of job and worker characteristics. UC Berkeley Labor Center. 2020 [cited 2020 Aug 18]. https://laborcenter.berkeley.edu/front-line-essential-jobs-in-california-a-profile-of-job-and-worker-characteristics/

[31] United States Department of Agriculture. Farm labor. 2020 [cited 2020 Jun 15]. https://www.ers.usda.gov/topics/farm-economy/farm-labor/#demographic

[32] United States Census Bureau. Five-year public use microdata sample (PUMS), 2014–2018. 2020 [cited 2020 Jun 15]. https://www.census.gov/programs-surveys/acs/technical-documentation/pums/documentation.html

[33] United States Census Bureau. 2010 census summary file 1. 2016 [cited 2020 Jun 5]. https://www.census.gov/data/datasets/2010/dec/summary-file-1.html<eref[REMOVED IF= FIELD]></eref>

[34] Chow NA, Griffin DW, Barker BM, Loparev VN, Litvintseva AP. Molecular detection of airborne *Coccidioides* in Tucson, Arizona. Med Mycol. 2016;54:584–92. 10.1093/mmy/myw022 PMC4962330 27143633

[35] Pappagianis D, Einstein H. Tempest from Tehachapi takes toll or *Coccidioides* conveyed aloft and afar. West J Med. 1978;129:527–30. PMC1238466 735056

[36] Das R, McNary J, Fitzsimmons K, Dobraca D, Cummings K, Mohle-Boetani J, et al. Occupational coccidioidomycosis in California: outbreak investigation, respirator recommendations, and surveillance findings. J Occup Environ Med. 2012;54:564–71. 22504958 10.1097/JOM.0b013e3182480556

[37] Cummings KC, McDowell A, Wheeler C, McNary J, Das R, Vugia DJ, et al. Point-source outbreak of coccidioidomycosis in construction workers. Epidemiol Infect. 2010;138:507–11. 10.1017/S0950268809990999 19845993

[38] Petersen LR, Marshall SL, Barton C, Hajjeh RA, Lindsley MD, Warnock DW, et al. Coccidioidomycosis among workers at an archeological site, northeastern Utah. Emerg Infect Dis. 2004;10:637–42. 10.3201/eid1004.030446 PMC3323065 15200853

[39] Standaert SM, Schaffner W, Galgiani JN, Pinner RW, Kaufman L, Durry E, et al. Coccidioidomycosis among visitors to a *Coccidioides immitis*–endemic area: an outbreak in a military reserve unit. J Infect Dis. 1995;171:1672–5. 10.1093/infdis/171.6.1672 7769316

[40] Wilken JA, Sondermeyer G, Shusterman D, McNary J, Vugia DJ, McDowell A, et al. Coccidioidomycosis among workers constructing solar power farms, California, USA, 2011–2014. Emerg Infect Dis. 2015;21:1997–2005. 10.3201/eid2111.150129 PMC4622237 26484688

[41] de Perio MA, Materna BL, Sondermeyer Cooksey GL, Vugia DJ, Su CP, Luckhaupt SE, et al. Occupational coccidioidomycosis surveillance and recent outbreaks in California. Med Mycol. 2019;57:S41–5. 10.1093/mmy/myy031 30690596

[42] Gorris ME, Cat LA, Zender CS, Treseder KK, Randerson JT. Coccidioidomycosis dynamics in relation to climate in the southwestern United States. Geohealth. 2018;2:6–24. 10.1002/2017GH000095 PMC7007142 32158997

[43] Schneider E, Hajjeh RA, Spiegel RA, Jibson RW, Harp EL, Marshall GA, et al. A coccidioidomycosis outbreak following the Northridge, Calif, earthquake. JAMA. 1997;277:904–8. 9062329

[44] Sondermeyer Cooksey GL, Wilken JA, McNary J, Gilliss D, Shusterman D, Materna BL, et al. Dust exposure and coccidioidomycosis prevention among solar power farm construction workers in California. Am J Public Health. 2017;107:1296–303. 10.2105/AJPH.2017.303820 28640687 PMC5508138

[45] Galgiani JN, Ampel NM, Blair JE, Catanzaro A, Johnson RH, Stevens DA, et al.; Infectious Diseases Society of America. Coccidioidomycosis. Clin Infect Dis. 2005;41:1217–23. 10.1086/496991 16206093

[46] Galgiani JN, Blair JE, Ampel NM, Thompson GR. Treatment for early, uncomplicated coccidioidomycosis: what is success? Clin Infect Dis. 2020;70:2008–12. 10.1093/cid/ciz933 31544210

[47] Valdivia L, Nix D, Wright M, Lindberg E, Fagan T, Lieberman D, et al. Coccidioidomycosis as a common cause of community-acquired pneumonia. Emerg Infect Dis. 2006;12:958–62. 10.3201/eid1206.060028 16707052 PMC3373055

[48] Chang DC, Anderson S, Wannemuehler K, Engelthaler DM, Erhart L, Sunenshine RH, et al. Testing for coccidioidomycosis among patients with community-acquired pneumonia. Emerg Infect Dis. 2008;14:1053–9. 10.3201/eid1407.070832 18598625 PMC2600364

[49] Tsang CA, Anderson SM, Imholte SB, Erhart LM, Chen S, Park BJ, et al. Enhanced surveillance of coccidioidomycosis, Arizona, USA, 2007-2008. Emerg Infect Dis. 2010;16:1738–44. 10.3201/eid1611.100475 21029532 PMC3294516

[50] Fu L, Wang B, Yuan T, Chen X, Ao Y, Fitzpatrick T, et al. Clinical characteristics of coronavirus disease 2019 (COVID-19) in China: a systematic review and meta-analysis. J Infect. 2020;80:656–65. 10.1016/j.jinf.2020.03.041 PMC7151416 32283155

[51] Goyal P, Choi JJ, Pinheiro LC, Schenck EJ, Chen R, Jabri A, et al. Clinical characteristics of Covid-19 in New York City. N Engl J Med. 2020;382:2372–4. 10.1056/NEJMc2010419 PMC7182018 32302078

