# Supplementary material for: Coccidioidomycosis and COVID-19 Co-Infection, United States, 2020
Source: Emerg Infect Dis. 2021 May;27(5):1266–73. doi: 10.3201/eid2705.204661 (PMC8084485; doi:10.3201/eid2705.204661)
Supplement: Appendix — Further information on results and additional references in a study of coccidiomycosis and COVID-19 co-infection. [file 20-4661-Techapp-s1.pdf]

Article DOI: https://doi.org/10.3201/eid2705.204661

Coccidioidomycosis and COVID-19 Co-Infection, United States, 2020

Appendix

Appendix Results

Coronavirus Disease Among Construction and Agricultural Workers

Construction, agriculture, and wildland firefighting are considered essential occupations under coronavirus disease (COVID-19) shelter-in-place guidelines for California and Arizona (*119*). Continued in-person work in these sectors poses challenges to maintaining physical distance and limiting contacts, resulting in higher risks for infection with severe acute respiratory syndrome coronavirus 2 (SARS-CoV-2) (*120*,*121*). For instance, ≈8% of persons in construction occupations across the United States are exposed to SARS-CoV-2 ≥1 time a month (*121*), and nearly 60% of the construction labor force in the United States has ≥1 factor (>65 years of age or preexisting medical condition) that is associated with higher risk for severe illness from COVID-19 (*122*). Outbreaks of COVID-19 have been documented at multiple construction sites across the United States (*123*–*126*), including a cluster of 10 confirmed cases and >30 exposures at a construction site in Santa Clara, California (*126*), and 75 confirmed cases among a construction crew in Salt Lake City, Utah (*125*).

Agricultural workers might also have heightened risk for COVID-19 because of high workforce mobility, shared transportation, and overcrowded living quarters, often shared with other workers, multigenerational families, or both (*127*–*129*). One study estimated that >133,000 agricultural workers across the United States had tested positive for COVID-19 by September 1, 2020 (*130*), and media reports have documented clusters of COVID-19 at farms in >17 states (*131*). Some of the largest COVID-19 clusters among agricultural workers have been documented in California counties to which coccidioidomycosis is endemic, including Merced (1 cluster involving 392 COVID-19 cases and 8 deaths) (*132*), Ventura (3 clusters with 201, 35, and 28 COVID-19 cases) (*133*–*135*), and Monterey (1 cluster involving 247 COVID-19 cases) (*136*).

COVID-19 within Carceral Facilities

Crowding, unsanitary conditions, and poor ventilation in prison environments is known to contribute to rapid spread of communicable respiratory diseases (*137*), including influenza and tuberculosis (*138–141*). As the introduction of 1 case of influenza into a prison setting has been found to be sufficient to spark a large outbreak (*139*), concerns surrounding the spread of COVID-19 among detainees and staff members at correctional facilities are high (*142*,*143*). During January 21–April 21, 2020, 82% (32/37) of reporting state and territorial health department jurisdictions reported confirmed COVID-19 cases among incarcerated or detained persons or staff members (*144*). As of April 21, 2020, a total of 4,893 cases and 88 deaths among incarcerated and detained persons and 2,778 cases and 15 deaths among staff members in these 37 jurisdictions have been reported to the Centers for Disease Control and Prevention (CDC) (*144*). Large COVID-19 outbreaks have been documented among incarcerated persons and staff working at carceral facilities in Lompoc Prison Complex in Lompoc, California (n = 1,111 cases) (*145*), San Quentin State Prison in California (n = 2,221) (*145*), Rikers Island in New York, New York (n = 1,711) (*146*), the Cook County Jail in Chicago, Illinois (n = 1,040) (*147*), and Marion Correctional Institution in Marion, Ohio (n = 2,168) (*148*). COVID-19 outbreaks have also been documented at training camps for fire-fighting crews comprising incarcerated persons in northern California (*149*).

Coccidioidomycosis within Carceral Facilities

In 1 review, 5/47 (11%) reported coccidioidomycosis outbreaks were among incarcerated populations (*150*). During 2007–2011, 19% of coccidioidomycosis cases in California were among incarcerated persons (*151*). Over a quarter of California Department of Corrections and Rehabilitation facilities, including Lompoc Prison Complex, where a COVID-19 outbreak of >1,000 cases occurred (*147*), are in regions with high coccidioidomycosis incidence (*152*). One study showed that *Coccidioides* spores were detected in 15% of air samples taken outside Avenal State Prison (*153*) in Kings County, California, where the incidence of coccidioidomycosis during 2007–2011 was nearly 6 times higher than that of the nearby city (2,195 vs. 411 cases/100,000 population) and 14 times higher than the surrounding county (155 cases/100,000) (*151*). Pleasant Valley State Prison in Fresno County, CA, recorded an incidence of 3,323 cases/100,000 persons in 2005, or 415 times higher than the incidence of the surrounding county (8 cases/100,000 persons) (*154*). Other prisons in endemic areas, such as those in 3 Kern County

cities of Delano, Wasco, and Taft, have reported incidence rates ≈2 times that of the surrounding county (*151*).

COVID-19 and Particulate Matter

Persons living in environments with high concentrations of dust, which is an important constituent of particulate matter with diameter ≤10 µm (PM10) or ≤2.5 µm (PM2.5), might be at elevated risk for infection with *Coccidioides* and SARS-CoV-2, as well as increased severity of COVID-19 infection. Exposure to PM10 and PM2.5 has been recognized as a risk factor for disease and death from viral respiratory infection (*155*), including severe acute respiratory syndrome coronavirus (*156*). Macrophages laden with fine particles might have reduced ability to induce immune responses leading to increased disease severity (*157*,*158*), and PM2.5 has been shown to exacerbate underlying health conditions such as diabetes and chronic lung disease (*159*), that can complicate the course of viral respiratory infections. Evidence from several countries suggests that both acute and chronic exposure to fine particulate matter is associated with increased COVID-19 disease and death (*160*). For example, 1 study found that a 1 $\mu g/m^3$ increase in long-term exposure to PM2.5 was associated with an 8% increase in the COVID-19 death rate in counties across the United States (*161*). In a study of 120 cities in China, elevated particulate matter in the previous 2 weeks was associated with a 2.2% increase in daily confirmed COVID-19 cases (*162*). In Italy, the number of days in the previous 4 years that had exceeded regulatory limits for atmospheric pollutants such as PM2.5 and PM10 was significantly associated with increased COVID-19 cases (*163*).

Diagnostic Tests for Coccidioidomycosis

Laboratory confirmation is necessary to distinguish coccidioidomycosis from other conditions (*45*), and serologic detection of anticoccidiodal antibodies is the most common method to diagnose coccidioidomycosis infection, partly because of the low sensitivity (≈46%–67%) of culture-based methods in respiratory samples (*164–166*). Newer serologic assays for IgM antibodies have sensitivities ranging from 68% to 88% (*167*,*168*), but IgM antibodies are not typically detectable until 7–21 days after symptom onset, and IgG antibodies even later (*164*,*165*,*169*). As a result, a coccidioidomycosis diagnosis might occur ≥1 month after symptom onset. The high rate of false negatives (≤32%) in coccidioidomycosis testing (*165*) further complicate the situation. Given the low sensitivities, experts recommend repeated testing if the original test is negative and symptoms persist (*98*,*170*). COVID-19 might make patients less

likely to return to their healthcare providers for a second test due to fear of exposure to SARS-CoV-2 in medical facilities.

Prevalence of Severe Disease in COVID-19 and Coccidioidomycosis Patients

Among coccidioidomycosis patients, ≈5% develop severe chronic infections, and 1% progress to disseminated disease in which the infection spreads beyond the pulmonary system (*68*). Disseminated disease can lead to meningitis, bone and skin lesions, swollen joints, hospitalization, and death (*56*). An estimated 80% of COVID-19 cases are mild (no or mild pneumonia), 15% are severe (severe pneumonia and respiratory distress), and 5% are critical (respiratory failure, septic shock, organ dysfunction/failure) (*82*).

COVID-19 Risk among Black/African American Persons

Growing evidence points toward higher risk for severe disease and death from COVID-19 among Black persons living in the United States. Compared with White persons, the age-adjusted COVID-19 death rate on August 4, 2020 was 3.7 times higher among those who identify as Black (80.4 deaths/100,000 persons vs. 35.9 deaths/100,000 persons) (*171*). This trend is reflected across coccidioidomycosis endemic states, such as California and Arizona. In Arizona, the age-adjusted death rate from COVID-19 among Black populations is 2.1 times higher than among White populations. In California, the age-adjusted death rate from COVID-19 among Black populations is 3.0 times higher than among White populations (*171*).

COVID-19 and Cloth Masks

The CDC has recommended that all Americans wear cloth face coverings in public settings to reduce transmission of SARS-CoV-2 (*99*). Although cloth masks are inadequate for filtering out the SARS-CoV-2 virus (which is only 70–90 nm in size) and do not protect the wearer from inhaling viral particles (*172*), masks can protect wearers from virus suspended in large droplets and can prevent wearers from spreading droplets by coughing, sneezing, or breathing. Mask use is recommended for the entire population because persons are believed to be most infectious before the onset of COVID-19 symptoms (*173*). Models suggest that if 80% of the American population wore cloth face masks consistently, COVID-19 transmission would decrease significantly, even if lock-down restrictions were loosened (D. Kai, unpub. data, https://arxiv.org/abs/2004.13553v1), but 1 national survey found that only 60% of respondents follow CDC mask recommendations (*174*).

References

52. Blair JE, Chang Y-HH, Cheng M-R, Vaszar LT, Vikram HR, Orenstein R, et al. Characteristics of patients with mild to moderate primary pulmonary coccidioidomycosis. Emerg Infect Dis. 2014;20:983–90. PubMed https://doi.org/10.3201/eid2006.131842

53. Shah AS, Heidari A, Civelli VF, Sharma R, Clark CS, Munoz AD, et al. The coincidence of 2 epidemics, coccidioidomycosis and SARS-CoV-2: a case report. J Investig Med High Impact Case Rep. 2020;8:2324709620930540. PubMed https://doi.org/10.1177/2324709620930540

54. Benedict K, Kobayashi M, Garg S, Chiller T, Jackson BR. Symptoms in blastomycosis, coccidioidomycosis, and histoplasmosis versus other respiratory illnesses in commercially insured adult outpatients—United States, 2016–2017. Clin Infect Dis. 2020;ciaa1554. https://doi.org/10.1093/cid/ciaa1554

55. De Filippo O, D’Ascenzo F, Angelini F, Bocchino PP, Conrotto F, Saglietto A, et al. Reduced rate of hospital admissions for ACS during Covid-19 outbreak in Northern Italy. N Engl J Med. 2020;383:88–9. PubMed https://www.nejm.org/doi/10.1056/NEJMc2009166

56. Rosenstein NE, Emery KW, Werner SB, Kao A, Johnson R, Rogers D, et al. Risk factors for severe pulmonary and disseminated coccidioidomycosis: Kern County, California, 1995–1996. Clin Infect Dis. 2001;32:708–14. PubMed https://doi.org/10.1086/319203

57. Sondermeyer Cooksey GL, Kamali A, Vugia D, Jain S. 1708. Epidemiology of coccidioidomycosis-associated hospitalizations and in-hospital deaths, California, 2000–2017. Open Forum Infect Dis. 2019;6:S626. https://doi.org/10.1093/ofid/ofz360.2512

58. Sondermeyer GL, Lee LA, Gilliss D, Vugia DJ. Coccidioidomycosis-associated deaths in California, 2000–2013. Public Health Rep. 2016;131:531–5. PubMed https://doi.org/10.1177/0033354916662210

59. CDC COVID-19 Response Team. Severe outcomes among patients with coronavirus disease 2019 (COVID-19)—United States, February 12–March 16, 2020. MMWR Morb Mortal Wkly Rep. 2020;69:343–6. PubMed https://doi.org/10.15585/mmwr.mm6912e2

60. Shi Y, Yu X, Zhao H, Wang H, Zhao R, Sheng J. Host susceptibility to severe COVID-19 and establishment of a host risk score: findings of 487 cases outside Wuhan. Crit Care. 2020;24:108. PubMed https://doi.org/10.1186/s13054-020-2833-7

61. Masannat FY, Ampel NM. Coccidioidomycosis in patients with HIV-1 infection in the era of potent antiretroviral therapy. Clin Infect Dis. 2010;50:1–7. PubMed https://doi.org/10.1086/648719

62. Blair JE, Smilack JD, Caples SM. Coccidioidomycosis in patients with hematologic malignancies. Arch Intern Med. 2005;165:113–7. PubMed https://doi.org/10.1001/archinte.165.1.113

63. Santelli AC, Blair JE, Roust LR. Coccidioidomycosis in patients with diabetes mellitus. Am J Med. 2006;119:964–9. PubMed https://doi.org/10.1016/j.amjmed.2006.03.033

64. Yang J, Zheng Y, Gou X, Pu K, Chen Z, Guo Q, et al. Prevalence of comorbidities and its effects in patients infected with SARS-CoV-2: a systematic review and meta-analysis. Int J Infect Dis. 2020;94:91–5. https://doi.org/10.1016/j.ijid.2020.03.017

65. Guo W, Li M, Dong Y, Zhou H, Zhang Z, Tian C, et al. Diabetes is a risk factor for the progression and prognosis of COVID-19. Diabetes Metab Res Rev. 2020;36:e3319. PubMed https://doi.org/10.1002/dmrr.3319

66. Fang L, Karakiulakis G, Roth M. Are patients with hypertension and diabetes mellitus at increased risk for COVID-19 infection? Lancet Respir Med. 2020;8:e21. PubMed https://doi.org/10.1016/S2213-2600(20)30116-8

67. Collaborative TO, Williamson E, Walker AJ, Bhaskaran KJ, Bacon S, Bates C, et al. OpenSAFELY: factors associated with COVID-19-related hospital death in the linked electronic health records of 17 million adult NHS patients. Nature. 2020;584:430–6. PubMed https://doi.org/10.1038/s41586-020-2521-4

68. Brown J, Benedict K, Park BJ, Thompson GR III. Coccidioidomycosis: epidemiology. Clin Epidemiol. 2013;5:185–97. PubMed

69. Stewart ER, Thompson GR III. Update on the epidemiology of coccidioidomycosis. Curr Fungal Infect Rep. 2016;10:141–6. https://doi.org/10.1007/s12281-016-0266-1

70. Louie L, Ng S, Hajjeh R, Johnson R, Vugia D, Werner SB, et al. Influence of host genetics on the severity of coccidioidomycosis. Emerg Infect Dis. 1999;5:672–80. PubMed https://doi.org/10.3201/eid0505.990508

71. Salehi S, Reddy S, Gholamrezanezhad A. Long-term pulmonary consequences of coronavirus disease 2019 (COVID-19). J Thorac Imaging. 2020;35:W87–9. PubMed https://doi.org/10.1097/RTI.0000000000000534

72. Bialek S, Boundy E, Bowen V, Chow N, Cohn A, Dowling N, et al.; CDC COVID-19 Response Team. Severe outcomes among patients with coronavirus disease 2019 (COVID-19)—United States, February 12–March 16, 2020. MMWR Morb Mortal Wkly Rep. 2020;69:343–6. https://doi.org/10.15585/mmwr.mm6912e2

73. Guo W, Li M, Dong Y, Zhou H, Zhang Z, Tian C, et al. Diabetes is a risk factor for the progression and prognosis of COVID-19. Diabetes Metab Res Rev. 2020;e3319. PubMed https://doi.org/10.1002/dmrr.3319

74. Santelli AC, Blair JE, Roust LR. Coccidioidomycosis in patients with diabetes mellitus. Am J Med. 2006;119:964–9. PubMed https://doi.org/10.1016/j.amjmed.2006.03.033

75. Geerlings SE, Hoepelman AI. Immune dysfunction in patients with diabetes mellitus (DM). FEMS Immunol Med Microbiol. 1999;26:259–65. PubMed https://doi.org/10.1111/j.1574-695X.1999.tb01397.x

76. The RECOVERY Collaborative Group. Dexamethasone in hospitalized patients with Covid-19—preliminary report. N Engl J Med. 2020 Jul 27 [Epub ahead of print]. PubMed https://doi.org/10.1056/NEJMoa2021436

77. Williamson EJ, Walker AJ, Bhaskaran K, Bacon S, Bates C, Morton CE, et al. Factors associated with COVID-19–related death using OpenSAFELY. Nature. 2020;584:430–6. PubMed https://doi.org/10.1038/s41586-020-2521-4

78. Fung M, Babik JM. COVID-19 in immunocompromised hosts: what we know so far. Clin Infect Dis. 2020;72:340–50. PubMed https://doi.org/10.1093/cid/ciaa863

79. Smith CE, Beard RR. Varieties of coccidioidal infection in relation to the epidemiology and control of the diseases. Am J Public Health Nations Health. 1946;36:1394–402. PubMed https://doi.org/10.2105/AJPH.36.12.1394

80. Vardavas CI, Nikitara K. COVID-19 and smoking: a systematic review of the evidence. Tob Induc Dis. 2020;18:20. PubMed https://doi.org/10.18332/tid/119324

81. CDC COVID-19 Response Team. Preliminary estimates of the prevalence of selected underlying health conditions among patients with coronavirus disease 2019—United States, February 12–March 28, 2020. MMWR Morb Mortal Wkly Rep. 2020;69:382–6.

82. Wu Z, McGoogan JM. Characteristics of and important lessons from the coronavirus disease 2019 (COVID-19) outbreak in China. JAMA. 2020;323:1239–42. PubMed https://doi.org/10.1001/jama.2020.2648

83. Zhou F, Yu T, Du R, Fan G, Liu Y, Liu Z, et al. Clinical course and risk factors for mortality of adult inpatients with COVID-19 in Wuhan, China: a retrospective cohort study. Lancet. 2020;395:1054–62. PubMed https://doi.org/10.1016/S0140-6736(20)30566-3

84. Sarosi GA, Parker JD, Doto IL, Tosh FE. Chronic pulmonary coccidioidomycosis. N Engl J Med. 1970;283:325–9. PubMed https://doi.org/10.1056/NEJM197008132830701

85. Twarog M, Thompson GR III. Coccidioidomycosis: recent updates. Semin Respir Crit Care Med. 2015;36:746–55. PubMed https://doi.org/10.1055/s-0035-1562900

86. Bercovitch RS, Catanzaro A, Schwartz BS, Pappagianis D, Watts DH, Ampel NM. Coccidioidomycosis during pregnancy: a review and recommendations for management. Clin Infect Dis. 2011;53:363–8. PubMed https://doi.org/10.1093/cid/cir410

87. Cha JM, Jung S, Bahng HS, Lim CM, Han DJ, Woo JH, et al. Multi-organ failure caused by reactivated coccidioidomycosis without dissemination in a patient with renal transplantation. Respirology. 2000;5:87–90. PubMed https://doi.org/10.1046/j.1440-1843.2000.00232.x

88. Keckich DW, Blair JE, Vikram HR, Seville MT, Kusne S. Reactivation of coccidioidomycosis despite antifungal prophylaxis in solid organ transplant recipients. Transplantation. 2011;92:88–93. PubMed https://doi.org/10.1097/TP.0b013e31821c1df6

89. Vucicevic D, Carey EJ, Blair JE. Coccidioidomycosis in liver transplant recipients in an endemic area. Am J Transplant. 2011;11:111–9. PubMed https://doi.org/10.1111/j.1600-6143.2010.03328.x

90. Mendoza N, Noel P, Blair JE. Diagnosis, treatment, and outcomes of coccidioidomycosis in allogeneic stem cell transplantation. Transpl Infect Dis. 2015;17:380–8. PubMed https://doi.org/10.1111/tid.12372

91. Blair JE. Approach to the solid organ transplant patient with latent infection and disease caused by *Coccidioides* species. Curr Opin Infect Dis. 2008;21:415–20. PubMed https://doi.org/10.1097/QCO.0b013e3283073828

92. Blair JE, Kusne S, Carey EJ, Heilman RL. The prevention of recrudescent coccidioidomycosis after solid organ transplantation. Transplantation. 2007;83:1182–7. PubMed https://doi.org/10.1097/01.tp.0000260143.54103.0d

93. Stockamp NW, Thompson GR III. Coccidioidomycosis. Infect Dis Clin North Am. 2016;30:229–46. PubMed https://doi.org/10.1016/j.idc.2015.10.008

94. Bercovitch RS, Catanzaro A, Schwartz BS, Pappagianis D, Watts DH, Ampel NM. Coccidioidomycosis during pregnancy: a review and recommendations for management. Clin Infect Dis. 2011;53:363–8. PubMed https://doi.org/10.1093/cid/cir410

95. Qin C, Zhou L, Hu Z, Zhang S, Yang S, Tao Y, et al. Dysregulation of immune response in patients with coronavirus 2019 (COVID-19) in Wuhan, China. Clin Infect Dis. 2020;71:762–8. PubMed https://doi.org/10.1093/cid/ciaa248

96. Beaman L, Benjamini E, Pappagianis D. Activation of macrophages by lymphokines: enhancement of phagosome-lysosome fusion and killing of *Coccidioides immitis*. Infect Immun. 1983;39:1201–7. PubMed https://doi.org/10.1128/IAI.39.3.1201-1207.1983

97. The RECOVERY Collaborative Group. Dexamethasone in hospitalized patients with Covid-19—preliminary report. N Engl J Med. 2021;384:693–704. PubMed https://doi.org/10.1056/NEJMoa2021436

98. Galgiani JN, Ampel NM, Blair JE, Catanzaro A, Geertsma F, Hoover SE, et al. 2016 Infectious Diseases Society of America (IDSA) clinical practice guideline for the treatment of coccidioidomycosis. Clin Infect Dis. 2016;63:e112–46. PubMed https://doi.org/10.1093/cid/ciw360

99. Centers for Disease Control and Prevention. Recommendation regarding the use of cloth face coverings, especially in areas of significant community-based transmission. 2020 [cited 2020 Aug 18]. https://www.cdc.gov/coronavirus/2019-ncov/prevent-getting-sick/cloth-face-cover.html

100. Akram SM, Koirala J. Coccidioidomycosis. 2020 [cited 2020 May 28]. https://www.ncbi.nlm.nih.gov/books/NBK448161/

101. Konda A, Prakash A, Moss GA, Schmoldt M, Grant GD, Guha S. Aerosol filtration efficiency of common fabrics used in respiratory cloth masks. ACS Nano. 2020;14:6339–47. PubMed https://doi.org/10.1021/acsnano.0c03252

102. McCullough NV, Brosseau LM, Vesley D. Collection of three bacterial aerosols by respiratory and surgical mask filters under varying conditions of flow and relative humidity. Ann Occup Hyg. 1997;41:677–90. PubMed https://doi.org/10.1016/S0003-4878(97)00022-7

103. State of California Department of Industrial Relations. Protection from valley fever. 2017 [cited 2020 May 28]. https://www.dir.ca.gov/dosh/valley-fever-home.html

104. Shaman J, Kohn M. Absolute humidity modulates influenza survival, transmission, and seasonality. Proc Natl Acad Sci U S A. 2009;106:3243–8. PubMed https://doi.org/10.1073/pnas.0806852106

105. Lowen AC, Steel J. Roles of humidity and temperature in shaping influenza seasonality. J Virol. 2014;88:7692–5. PubMed https://doi.org/10.1128/JVI.03544-13

106. Lowen AC, Mubareka S, Steel J, Palese P. Influenza virus transmission is dependent on relative humidity and temperature. PLoS Pathog. 2007;3:1470–6. PubMed https://doi.org/10.1371/journal.ppat.0030151

107. Shaman J, Pitzer VE, Viboud C, Grenfell BT, Lipsitch M. Absolute humidity and the seasonal onset of influenza in the continental United States [Erratum in: PLoS Biol. 2010;8:10.1371/annotation/35686514-b7a9-4f65-9663-7baefc0d63c0]. PLoS Biol. 2010;8:e1000316. PubMed https://doi.org/10.1371/journal.pbio.1000316

108. Baker RE, Mahmud AS, Wagner CE, Yang W, Pitzer VE, Viboud C, et al. Epidemic dynamics of respiratory syncytial virus in current and future climates. Nat Commun. 2019;10:5512. PubMed https://doi.org/10.1038/s41467-019-13562-y

109. Pitzer VE, Viboud C, Alonso WJ, Wilcox T, Metcalf CJ, Steiner CA, et al. Environmental drivers of the spatiotemporal dynamics of respiratory syncytial virus in the United States. PLoS Pathog. 2015;11:e1004591. PubMed https://doi.org/10.1371/journal.ppat.1004591

110. Martinez ME. The calendar of epidemics: seasonal cycles of infectious diseases. PLoS Pathog. 2018;14:e1007327. PubMed https://doi.org/10.1371/journal.ppat.1007327

111. Wang J, Tang K, Feng K, Lv W. Impact of temperature and relative humidity on the transmission of COVID-19: a modeling study in China and the United States. SSRN Electron J. 2020. https://dx.doi.org/10.2139/ssrn.3551767

112. Sajadi MM, Habibzadeh P, Vintzileos A, Shokouhi S, Miralles-Wilhelm F, Amoroso A. Temperature, humidity, and latitude analysis to estimate potential spread and seasonality of coronavirus disease 2019 (COVID-19). JAMA Netw Open. 2020;3:e2011834. PubMed https://doi.org/10.1001/jamanetworkopen.2020.11834

113. Ma Y, Zhao Y, Liu J, He X, Wang B, Fu S, et al. Effects of temperature variation and humidity on the death of COVID-19 in Wuhan, China. Sci Total Environ. 2020;724:138226. PubMed https://doi.org/10.1016/j.scitotenv.2020.138226

114. Baker RE, Yang W, Vecchi GA, Metcalf CJE, Grenfell BT. Susceptible supply limits the role of climate in the early SARS-CoV-2 pandemic. Science. 2020;369:315–9. PubMed https://doi.org/10.1126/science.abc2535

115. Csavina J, Field J, Félix O, Corral-Avitia AY, Sáez AE, Betterton EA. Effect of wind speed and relative humidity on atmospheric dust concentrations in semi-arid climates. Sci Total Environ. 2014;487:82–90. PubMed https://doi.org/10.1016/j.scitotenv.2014.03.138

116. Olson DR, Huynh M, Fine A, Baumgartner J, Castro A, Chan HT, et al.; New York City Department of Health and Mental Hygiene COVID-19 Response Team. Preliminary estimate of excess mortality during the COVID-19 outbreak—New York City, March 11–May 2, 2020. MMWR Morb Mortal Wkly Rep. 2020;69:603–5. PubMed https://doi.org/10.15585/mmwr.mm6919e5

117. Frank AL, Liebman AK, Ryder B, Weir M, Arcury TA. Health care access and health care workforce for immigrant workers in the agriculture, forestry, and fisheries sector in the southeastern US. Am J Ind Med. 2013;56:960–74. PubMed https://doi.org/10.1002/ajim.22183

118. McCurdy SA, Portillo-Silva C, Sipan CL, Bang H, Emery KW. Risk for coccidioidomycosis among Hispanic farm workers, California, USA, 2018. Emerg Infect Dis. 2020;26:1430–7. PubMed https://doi.org/10.3201/eid2607.200024

119. State of California. Essential workforce. 2020 [cited 2020 May 15]. https://covid19.ca.gov/essential-workforce/

120. Koh D. Occupational risks for COVID-19 infection. Occup Med (Lond). 2020;70:3–5. PubMed https://doi.org/10.1093/occmed/kqaa036

121. Baker MG, Peckham TK, Seixas NS. Estimating the burden of United States workers exposed to infection or disease: a key factor in containing risk of COVID-19 infection. PLoS One. 2020;15:e0232452. PubMed https://doi.org/10.1371/journal.pone.0232452

122. Brown S, Brooks R, Dong XS. Coronavirus and health disparities in construction. The Center for Construction Research and Training. 2020 [cited 2020 Oct 16]. https://www.cpwr.com/wp-content/uploads/publications/DataBulletin-May2020.pdf

123. Brown J. Coronavirus has struck construction sites across Colorado, including a school and off-campus housing project. The Colorado Sun. 2020 [cited 2020 Oct 16]. https://coloradosun.com/2020/07/24/coronavirus-at-construction-sites/

124. Reed J. "Significant outbreak" of COVID-19 among construction workers at Alabama's Bryant-Denny Stadium. MSN. 2020 [cited 2020 Oct 16]. https://www.msn.com/en-us/sports/ncaafb/significant-outbreak-of-covid-19-among-construction-workers-at-alabamas-bryant-denny-stadium/ar-BB14ntGN

125. Rischar H. COVID-19 outbreak among construction workers may delay opening of new SLC. Construction & Demolition Recycling. 2020 [cited 2020 Oct 16]. https://www.cdrecycler.com/article/covid-19-outbreak-construction-workers-delay-opening-slc/

126. Santa Clara County Public Health. COVID-19 cases at construction sites highlight need for continued vigilance in sectors that have reopened. 2020 [cited 2020 Oct 16]. https://www.sccgov.org/sites/covid19/Pages/press-release-06-12-2020-cases-at-construction-sites.aspx

127. Centers for Disease Control and Prevention. Communities, schools, workplaces, and events. 2020 [cited 2020 Oct 16]. https://www.cdc.gov/coronavirus/2019-ncov/community/guidance-agricultural-workers.html

128. Arcury TA, Weir M, Chen H, Summers P, Pelletier LE, Galván L, et al. Migrant farmworker housing regulation violations in North Carolina. Am J Ind Med. 2012;55:191–204. PubMed https://doi.org/10.1002/ajim.22011

129. Quandt SA, Brooke C, Fagan K, Howe A, Thornburg TK, McCurdy SA. Farmworker housing in the United States and its impact on health. New Solut. 2015;25:263–86. PubMed https://doi.org/10.1177/1048291115601053

130. Lusk J. Purdue food and agriculture vulnerability index. Purdue University. 2020 [cited 2020 Oct 16]. https://ag.purdue.edu:443/agecon/Pages/FoodandAgVulnerabilityIndex.aspx?_ga=2.49471334.1159720487.1600111458-250602208.1598985334

131. National Center for Farmworker Health, Inc. COVID-19 in rural America: impact on farms and agricultural workers. 2020 Oct [cited 2020 Oct 16]. http://www.ncfh.org/msaws-and-covid-19.html

132. Lauten-Scrivner A. Foster farms COVID-19 deaths among worst work-related outbreaks in California, official says. Merced Sun-Star. 2020 Sep 16 [cited 2020 Oct 16]. https://www.mercedsunstar.com/news/coronavirus/article245767575.html

133. Botts J, Cimini K. Investigation: COVID rips through motel rooms of guest workers who pick nation’s produce. CalMatters. 2020 [cited 2020 Oct 16]. https://calmatters.org/california-divide/2020/08/guest-worker-covid-outbreak-california/

134. de los Santos S. Several employees test positive for COVID-19 at produce packing site in Santa Paula. NewsChannel 3–12. 2020 May 14 [cited 2020 Oct 16]. https://keyt.com/health/coronavirus/2020/05/13/employee-tests-positive-for-covid-19-at-produce-packing-site-in-santa-paula/

135. Rode E. Farmworker housing outbreak: 154 workers released from isolation Tuesday. Ventura County Star. 2020 Jul 7 [cited 2020 Oct 16]. https://www.vcstar.com/story/news/local/2020/07/07/oxnard-farmworker-housing-covid-outbreak-coronavirus-recovery-rate/5387928002/

136. Walljasper C, Rosenberg M, Cooke K. Coronavirus spreads among fruit and vegetable packers, worrying U.S. officials. Reuters. 2020 [cited 2020 Oct 16]. https://www.reuters.com/article/us-health-coronavirus-usa-farmworkers-idUSKBN23I1FO

137. Simpson PL, Simpson M, Adily A, Grant L, Butler T. Prison cell spatial density and infectious and communicable diseases: a systematic review [Erratum in: BMJ Open. 2020;10: e026806corr1]. BMJ Open. 2019;9:e026806. PubMed https://doi.org/10.1136/bmjopen-2018-026806

138. Dolan K, Wirtz AL, Moazen B, Ndeffo-Mbah M, Galvani A, Kinner SA, et al. Global burden of HIV, viral hepatitis, and tuberculosis in prisoners and detainees. Lancet. 2016;388:1089–102. PubMed https://doi.org/10.1016/S0140-6736(16)30466-4

139. Stanley LL. Influenza at San Quentin Prison, California. Public Health Rep 1896–1970. 1919;34:996. https://www.jstor.org/stable/pdf/4575142.pdf

140. Besney J, Moreau D, Jacobs A, Woods D, Pyne D, Joffe AM, et al. Influenza outbreak in a Canadian correctional facility. J Infect Prev. 2017;18:193–8. PubMed https://doi.org/10.1177/1757177416689725

141. Finnie TJR, Copley VR, Hall IM, Leach S. An analysis of influenza outbreaks in institutions and enclosed societies. Epidemiol Infect. 2014;142:107–13. PubMed https://doi.org/10.1017/S0950268813000733

142. Akiyama MJ, Spaulding AC, Rich JD. Flattening the curve for incarcerated populations—Covid-19 in jails and prisons. N Engl J Med. N Engl J Med. 2020;2005687.

143. Kinner SA, Young JT, Snow K, Southalan L, Lopez-Acuña D, Ferreira-Borges C, et al. Prisons and custodial settings are part of a comprehensive response to COVID-19. Lancet Public Health. 2020;5:e188–9. PubMed https://doi.org/10.1016/S2468-2667(20)30058-X

144. Wallace M, Hagan L, Curran KG, Williams SP, Handanagic S, Bjork A, et al. COVID-19 in correctional and detention facilities—United States, February–April 2020. MMWR Morb Mortal Wkly Rep. 2020;69:587–90. PubMed https://doi.org/10.15585/mmwr.mm6919e1

145. California Department of Corrections and Rehabilitation. Population COVID-19 tracking. 2020 [cited 2020 Aug 6]. https://www.cdcr.ca.gov/covid19/population-status-tracking/

146. Correctional Health Services and Department of Correction. Board of Correction daily Covid-19 update. 2020 [cited 2020 May 21]. https://www1.nyc.gov/assets/boc/downloads/pdf/News/covid-19/Public_Reports/Board%20of%20Correction%20Daily%20Public%20Report_5_14_2020.pdf

147. The New York Times. Coronavirus in the U.S.: latest map and case count. 2020 [cited 2020 May 28]. https://www.nytimes.com/interactive/2020/us/coronavirus-us-cases.html?auth=login-email&login=email

148. Ohio Department of Rehabilitation & Correction. COVID-19 inmate testing. 2020 [cited 2020 May 21]. https://drc.ohio.gov/Portals/0/DRC%20COVID-19%20Information%2004-19-2020%20%201305.pdf

149. Sabalow R, Pohl J. California severely short on firefighting crews after COVID-19 lockdown at prison camps. The Sacramento Bee. 2020 [cited 2020 Aug 18]. https://www.sacbee.com/news/california/fires/article243977827.html

150. Freedman M, Jackson BR, McCotter O, Benedict K. Coccidioidomycosis outbreaks, United States and worldwide, 1940–2015. Emerg Infect Dis. 2018;24:417–23. PubMed https://doi.org/10.3201/eid2403.170623

151. MacLean M. Epidemiology of coccidioidomycosis—15 California counties, 2007–2011. 2014 [cited 2020 May 28]. https://www.vfce.arizona.edu/sites/vfce/files/the_epidemiology_of_coccidioidomycosis_collaborative_county_report.pdf

152. Prison Law Office. Valley fever and CDCR housing. 2019 [cited 2020 May 28]. https://prisonlaw.com/wp-content/uploads/2019/04/Valley-Fever-info-April-2019.pdf

153. Dubey T. Increased cases of valley fever disease in central California: an update. In: Razzaghi-Abyaneh M, Shams-Ghahfarokhi M, and Rai M, editors. Medical mycology: current trends and future prospects. Boca Raton (FL): CRC Press; 2015. p. 274–88.

154. Lee LA, Yuan J, Vugia D, Wheeler C, Chapnick R, Mohle-Boetani J. Increased coccidioidomycosis among inmates at a California prison. J Correct Health Care. 2017;23:347–52. PubMed https://doi.org/10.1177/1078345817716451

155. Ciencewicki J, Jaspers I. Air pollution and respiratory viral infection. Inhal Toxicol. 2007;19:1135–46. PubMed https://doi.org/10.1080/08958370701665434

156. Cui Y, Zhang ZF, Froines J, Zhao J, Wang H, Yu SZ, et al. Air pollution and case fatality of SARS in the People's Republic of China: an ecologic study. Environ Health. 2003;2:15. PubMed https://doi.org/10.1186/1476-069X-2-15

157. Becker S, Soukup JM. Exposure to urban air particulates alters the macrophage-mediated inflammatory response to respiratory viral infection. J Toxicol Environ Health A. 1999;57:445–57. PubMed https://doi.org/10.1080/009841099157539

158. Kaan PM, Hegele RG. Interaction between respiratory syncytial virus and particulate matter in guinea pig alveolar macrophages. Am J Respir Cell Mol Biol. 2003;28:697–704. PubMed https://doi.org/10.1165/rcmb.2002-0115OC

159. Zanobetti A, Schwartz J. Are diabetics more susceptible to the health effects of airborne particles? Am J Respir Crit Care Med. 2001;164:831–3. PubMed https://doi.org/10.1164/ajrccm.164.5.2012039

160. Brandt EB, Beck AF, Mersha TB. Air pollution, racial disparities, and COVID-19 mortality. J Allergy Clin Immunol. 2020;146:61–3. PubMed https://doi.org/10.1016/j.jaci.2020.04.035

161. Wu X, Nethery RC, Sabath BM, Braun D, Dominici F. Air pollution and COVID-19 mortality in the United States: strengths and limitations of an ecological regression analysis. Science Advances; 2020;6:eabd4049. PubMed https://doi.org/10.1126/sciadv.abd4049

162. Zhu Y, Xie J, Huang F, Cao L. Association between short-term exposure to air pollution and COVID-19 infection: evidence from China. Sci Total Environ. 2020;727:138704. PubMed https://doi.org/10.1016/j.scitotenv.2020.138704

163. Fattorini D, Regoli F. Role of the chronic air pollution levels in the Covid-19 outbreak risk in Italy. Environ Pollut. 2020;264:114732. PubMed https://doi.org/10.1016/j.envpol.2020.114732

164. Malo J, Holbrook E, Zangeneh T, Strawter C, Oren E, Robey I, et al. Enhanced antibody detection and diagnosis of coccidioidomycosis with the MiraVista IgG and IgM detection enzyme immunoassay. J Clin Microbiol. 2017;55:893–901. PubMed https://doi.org/10.1128/JCM.01880-16

165. Kassis C, Durkin M, Holbrook E, Myers R, Wheat L. Advances in diagnosis of progressive pulmonary and disseminated coccidioidomycosis. Clin Infect Dis. 2020;ciaa188. PubMed https://doi.org/10.1093/cid/ciaa188

166. Dizon D, Mitchell M, Dizon B, Libke R, Peterson MW. The utility of real-time polymerase chain reaction in detecting *Coccidioides immitis* among clinical specimens in the Central California San Joaquin Valley. Med Mycol. 2019;57:688–93. PubMed https://doi.org/10.1093/mmy/myy111

167. Blair JE, Coakley B, Santelli AC, Hentz JG, Wengenack NL. Serologic testing for symptomatic coccidioidomycosis in immunocompetent and immunosuppressed hosts. Mycopathologia. 2006;162:317–24. PubMed https://doi.org/10.1007/s11046-006-0062-5

168. Khan S, Saubolle MA, Oubsuntia T, Heidari A, Barbian K, Goodin K, et al. Interlaboratory agreement of coccidioidomycosis enzyme immunoassay from two different manufacturers. Med Mycol. 2019;57:441–6. PubMed https://doi.org/10.1093/mmy/myy059

169. Saubolle MA. Laboratory aspects in the diagnosis of coccidioidomycosis. Ann N Y Acad Sci. 2007;1111:301–14. PubMed https://doi.org/10.1196/annals.1406.049

170. Valley Fever Center for Excellence. Valley fever (coccidioidomycosis): tutorial for primary care professionals. 2016 [cited 2020 May 22]. https://vfce.arizona.edu/sites/vfce/files/tutorial_for_primary_care_professionals.pdf

171. APM Research Lab. COVID-19 deaths analyzed by race and ethnicity. 2020 [cited 2020 Aug 18]. https://www.apmresearchlab.org/covid/deaths-by-race

172. Kim JM, Chung YS, Jo HJ, Lee NJ, Kim MS, Woo SH, et al. Identification of coronavirus isolated from a patient in Korea with COVID-19. Osong Public Health Res Perspect. 2020;11:3–7. PubMed https://doi.org/10.24171/j.phrp.2020.11.1.02

173. He X, Lau EHY, Wu P, Deng X, Wang J, Hao X, et al. Temporal dynamics in viral shedding and transmissibility of COVID-19 [Erratum in: Nat Med. 2020;26:1491–3]. Nat Med. 2020;26:672–5. PubMed https://doi.org/10.1038/s41591-020-0869-5

174. Goldberg MH, Gustafson A, Maibach EW, Ballew MT, Bergquist P, Kotcher JE X, et al. Mask-wearing increased after a government recommendation: a natural experiment in the U.S. during the COVID-19 pandemic. Front. Commun. 2020;5:44. https://doi.org/10.3389/fcomm.2020.00044
